# Quercitrin restrains the growth and invasion of lung adenocarcinoma cells by regulating gap junction protein beta 2

**DOI:** 10.1080/21655979.2022.2037372

**Published:** 2022-02-23

**Authors:** Deng Yun Li, Li Xiao Yue, Shi Guang Wang, Tian Xiao Wang

**Affiliations:** a School of Medicine, Zhengzhou University of Industrial Technology, Zhengzhou, Henan, China; b School of Pharmacy, Henan University, Kaifeng, Henan, China

**Keywords:** Quercitrin, GJB2, LUAD, invasion

## Abstract

Lung adenocarcinoma (LUAD) is the most prevalent subtype of non-small cell lung cancer (NSCLC) with high lethality, and quercitrin exhibits anticancer characteristics. Here, we attempted to uncover the anticancer activity of quercitrin in LUAD. In this work, quercitrin prohibited the cell viability and clone-formation of LUAD cells *in vitro*. Meanwhile, quercitrin treatment reduced the aggressive phenotypes in LUAD cells. Further, Gap Junction Protein Beta 2 (GJB2) expression was aberrantly higher in LUAD when compared within control tissue. The higher expression of GJB2 is associated with an inferior overall survival for patients with LUAD. Finally, the reintroduction of GJB2 offset the inhibiting influence of quercitrin in LUAD cells. Altogether, these findings disclosed that quercitrin suppressed the growth and metastatic-related traits of LUAD cells partly *via* regulating GJB2 expression.

## Graphical abstract

**Figure:**
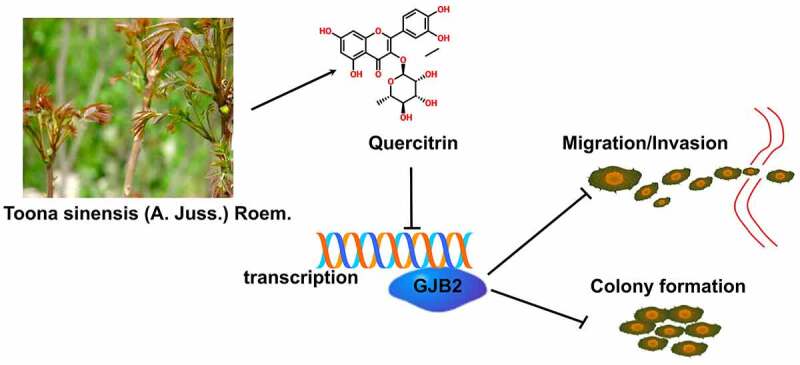

## Introduction

Despite the great advance in diagnostic strategy and targeted therapies, the clinical prognosis of patients with lung adenocarcinoma (LUAD) remains unsatisfactory owing to its recurrence and metastasis [1–3]. The metastatic procedure involves highly-complicated processes, including cancer cell migration, invasion, tumor angiogenesis, and epithelial-to-mesenchymal transition (EMT) [4]. As metastasis in clinical is the primary trigger of treatment failure and tumor relapse, blocking cancer metastasis will benefit anti-cancer therapy [5,6]. Targeting tumor cell invasion and migration has been demonstrated to be potential strategies for treating tumors [7]. Hence, it is imperative to exploit a novel treatment option for depressing cancer metastasis.

**Table 1.** The theoretical affinity of quercitrin with respect to GJB2

compound	D–H···A	Bond angle (θ)				distance (Å)
		XDA	DAY	DHA	HAY	
Quercitrin	A:TRP134:NE1 -:O	73.987	102.156			3.07447
	A:SER138:OG -:O	85.367	105.4			3.36529
	:H – A:SER138:OG			117.277	122.985	2.3308

*Toona sinensis* (Juss.) M.Roem. (syn. *Cedrela sinensis Juss*.) is an upland tree that has been used as nutritious food and traditional medicine in China [8]. *T. sinensis* leaves contain a wide variety of bioactive compounds, including methyl gallte, kaemferol, gallic acid, quercetin, quercitrin, etc [9]. Quercitrin (3-rhamnosyl quercetin), a glycoside of quercetin, has been found as the predominant bioactive ingredient in *T. sinensis* leaves [10,11]. Recent studies uncovered that quercitrin exhibits a scavenger and anti-oxidant role; consequently, quercitrin has become the center of attention for its potential anti-carcinogenic activity [12]. In non-small cell lung cancer (NSCLC), quercitrin-induced anti-proliferative action could inhibit the cell adhesion pathway, directing cancer cells to migration [13]. Nevertheless, no relevant reports intensely focus on the anti-cancerous properties of quercitrin in LUAD.

GJB2 (also known as connexin 26) is located on chromosome 13q12.11 and contains three exons. GJB2 is an oncogene associated with tumor growth, EMT, and metastasis in multiple types of cancer [14,15]. High expression of GJB2 was observed in patients with LUAD, and GJB2 may be a potentially valuable prognostic molecular biomarker of inferior survival in LUAD [16]. Mechanistically, the internalization of GJB2 facilitates the proliferation, epithelial-mesenchymal transition (EMT), and migration of non-small cell lung cancer (NSCLC) cells under hypoxic conditions by aberrant activation of the P53/MDM2 signaling pathway [17]. In addition, the PI3K/Akt signaling pathway connected to GJB2 is reported to cause acquired gefitinib resistance in NSCLC cells *via* activating EMT [18]. GJB2 also helps lung squamous cell carcinoma (SCC), acquire aggressive phenotypes, lymph node metastasis, and poor prognosis [19]. All these data suggested that aberrant GJB2 expression is associated with tumor progression in lung cancer.

Herein, we confirmed that quercitrin attenuated the proliferation, migration, and invasion ability of LUAD cells. GJB2 was markedly upregulated in LUAD, and GJB2 deficiency distinctly repressed the aggressive phenotypes of LUAD cells. Mechanistically, we disclosed that quercitrin impedes the cell growth and invasiveness of LUAD cells *via* inhibition of GJB2.

## Materials and methods

### Cell culture

H1299 and H1650 cells were purchased from ATCC (Rockville, USA). Cells were cultured in Dulbecco’s Modified Eagle Medium (DMEM) (Thermo Fisher Scientific, Waltham, MA, USA) supplemented with 10% Fetal Bovine Serum (FBS) at 5% CO_2_ and 37°C. Quercitrin (purity≥98%) was bought from Yuanye Bio-technology (Shanghai, China). 5-Fluorouracil (5-FU) was purchased from Selleck Chemicals (Houston, USA). Quercitrin or 5-FU was dissolved in dimethylsulfoxide (DMSO) (Sigma, Shanghai, China).

### Cell transfections

Two different siRNAs (GJB2 #1 and GJB2 #2) were constructed by GenePharma (Shanghai, China). The full length of GJB2 was synthesized and cloned into the pCDNA3.1 vector (Thermo Fisher Scientific), generating the pCDNA3.1-GJB2 plasmid (GJB2-OE). Cells were transfected with siRNA or plasmid using the Lipofectamine 3000 Reagent (Thermo Fisher Scientific).

### Cell proliferation

cells (1 × 10^3^) were plated into 96-well plates. After 24 h, cells were cultured with media containing quercitrin. After 24, 48, or 72 h, 10 μl of cell counting kit-8 (CCK-8, Beijing, China) solution was added. Finally, the optical density (OD) value was measured at 450 nm.

### Colony formation

Approximately 1000 cells were seeded into 6-well plates. The culture medium containing quercitrin in plates was replaced every three days. After two weeks, cell colonies were stained with crystal violet (1%). The number of cell colonies was counted under an inverted microscope.

### Invasion assay

The upper compartment of the Transwell chamber (8.0 μm; BD) was coated with Matrigel (BD). 5 × 10^3^ cells were seeded into the upper chamber and treated with quercitrin. The lower compartment was filled with 600 μl of medium supplement with 10% FBS. After 24 h, the invading cells were dyed by crystal violet (1%) and were counted through an inverted microscope.

### Migration analysis

Cells (5 × 10^5^) were seeded into 6-well plates. After the cells in plates reached 80% confluence, a straight wound was artificially made by utilizing a 100 μl pipette tip. Cells in plates were maintained with media containing quercitrin. Images were captured at 0 or 24 h.

### Molecular docking

The X-ray crystal structures of candidate proteins (ADAM12, PROM2, PSAT1, ANKRD22, KIF26B, CRABP2, THBS2, COL1A1, MMP11, LINC00673, HS6ST2, SIX1, ANLN, SULF1, COL10A1, CDCA7, CXCL14, CTHRC1, KIAA0101, TMPRSS4, COL11A1, SPP1, TOP2A, GREM1, GJB2, MMP1, MMP12) were obtained from RCSB Protein Data Bank (http://www.rcsb.org). Quercitrin was drawn by using ISIS-Draw. AutoDock (http://autodock.scripps.edu/) is designed to predict the intermolecular interaction energy between the ligand and the protein and the intra-molecular interaction energy of the ligand.

### Western blot

Immunoblotting was performed as described previously [20]. GJB2 and β-actin antibodies were purchased from Santa Cruz Biotechnology (Santa Cruz, CA, USA). Horseradish peroxidase (HRP)-linked anti-rabbit antibody was purchased from Biyuntian Biotechnology (Shanghai, China).

### Quantitative RT-PCR (qRT-PCR) assay

Total RNA was extracted using TRIZOL® reagent (Thermo Fisher Scientific), and cDNA synthesis was performed using a TaKaRa PrimeScript RT reagent kit (TaKaRa Biotechnology, Dalian, China). The real-time PCR was conducted using a SYBR®Premix Ex Taq™ Green I (TaKaRa) on Roche Light Cycler 480. GAPDH was used as an internal control. Primers were listed as follows: GJB2 forward, 5′-TCGCATTATGATCCTCGTTGTG-3′ and reverse, 5′-GGGGAAGTAGTGATCGTAGCAC-3′; GAPDH forward, 5′-TGTGGGCATCAATGGATTTGG-3′ and reverse, 5′-ACACCATGTATTCCGGGTCAAT-3′.

### Immunofluorescence

H1299 or H1650 cells in 24-well plates were fixed using 4% paraformaldehyde and blocked with 5% goat serum. Then, cells were incubated with GJB2 antibody (1:500, Abcam) overnight at 4°C. Subsequently, cells were incubated with a fluorochrome-conjugated anti-rabbit secondary antibody (1:1000, Bioworld, China) for 2 h. Subsequently, cells were stained with DAPI for 5 min. Images were captured under a fluorescence microscope.

### Statistical analysis

The data and half-maximal inhibitory concentration (IC50) values were calculated by GraphPad Prism 8.0 and shown as Mean ± Standard deviation (SD). Statistical analysis was determined using Student’s t-test or one-way ANOVA followed by Tukey’s post hoc test. *P*< 0.05 is statistically significant.

## Result

Here, we attempted to uncover the anticancer activity of quercitrin in LUAD. In this work, quercitrin suppressed the cell growth, migration, and invasion of LUAD cells *in vitro*. Further, the GJB2 level was higher in LUAD tissues when compared to normal tissue. Mechanistically, quercitrin suppressed the growth and metastatic-related traits of LUAD cells *via* regulating GJB2 expression.

### LUAD cells migration and invasion are suppressed by quercitrin

**Figure 1.**
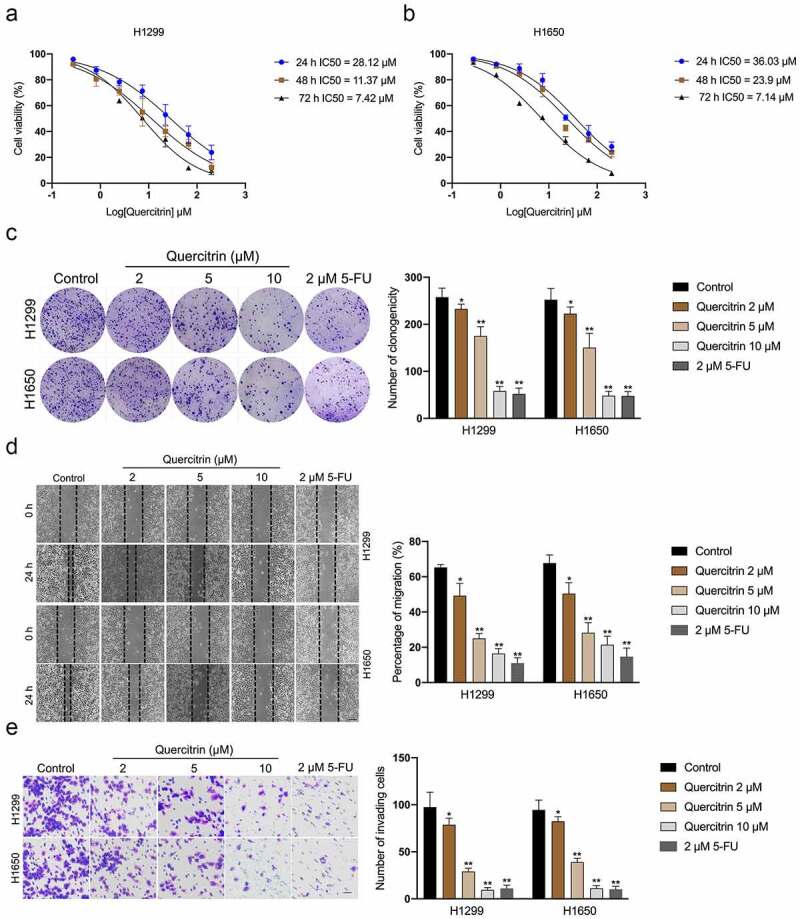
Quercitrin reduces LUAD cell growth, migration, and invasion. a-b. CCK-8 proliferation assay of H1299 and H1650 cells treated with different concentrations of quercitrin for 24 hours, 48 hours, or 72 h. c. Colony formation assay of H1299 and H1650 cells treated with quercitrin (2, 5, 10 μM). d. Wound healing assay of H1299 and H1650 cells treated with quercitrin. E. Transwell invasion assays showed that quercitrin decreased the invasion ability of H1299 and H1650 cells. Data represent the Mean ± SD for at least three independent experiments. *P< 0.05, **P< 0.01 compared with control.

Firstly, CCK-8 assays were performed to evaluate the effect of quercitrin on LUAD cells’ (H1299 and H1650) growth. As exhibited in Figure 1(a,b), the IC50 of quercitrin for H1299 was 28.12 μM at 24 h, and IC50 for H1650 was 36.03 μM at 24 h. To rule out the possibility that quercitrin suppression on cell proliferation was contributing to the effects on LUAD cells migration and invasion, we measured proliferation after 24 h of treatment with quercitrin. The IC50 of quercitrin in H1299 and H1650 cells was nearly 30 μM and one-third of the IC50 value (10 μM) as a starting concentration. No significant differences in the inhibitory ratio of LUAD cells treated with 2, 5, or 10 μM quercitrin for 24 h were observed compared with cells treated with vehicle (data not shown). Therefore, 2, 5, and 10 μML of quercitrin were used in the following assays. Similarly, quercitrin (2, 5, 10 μM) dramatically diminished the clonogenic capacities of LUAD cells *in vitro* (Figure 19(c)). 2 μM 5-Fluorouracil (5-FU) was used as the positive control [21]. We further evaluated whether quercitrin altered the migration and invasive features of LUAD cells. H1299 and H1650 cells were exposed to quercitrin, and the wound healing test was implemented. As indicated in Figure 1(d), quercitrin profoundly declined the migration capacity of H1299 and H1650 cells in a dose-dependent manner. Consistently, quercitrin also decreased the invasion capacity of LUAD cells (Figure 1(e)). H1299 xenograft model was employed to evaluate the antitumor potential of quercitrin *in vivo*. As shown in Supplementary Figure 1, smaller tumor volumes and lower tumor weights were observed in quercitrin-treated mice as compared with mice treated with vehicle. Collectively, these results imply that quercitrin impedes LUAD cells growth, migration, and invasion.

### GJB2 is upregulated in LUAD

**Figure 2.**
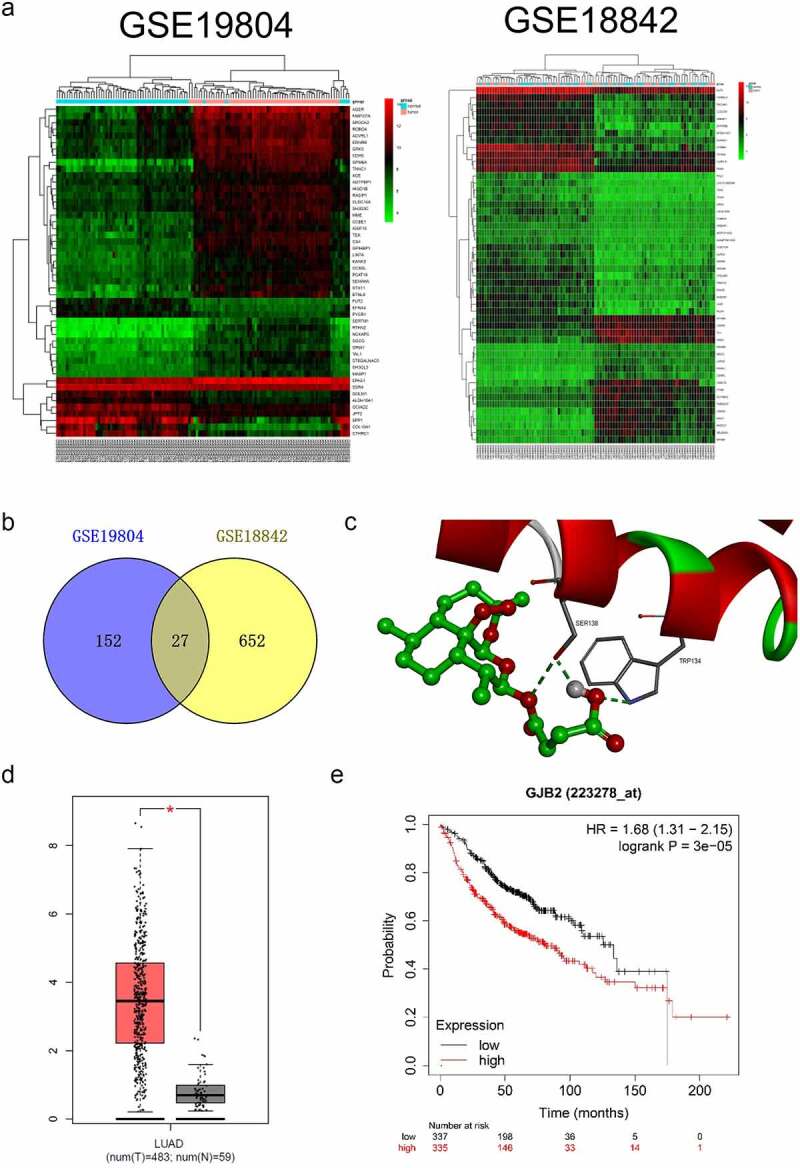
Differentially expressed genes in LUAD. a. Heat maps of dysregulated genes in GSE18842 and GSE19804 databases. b. Venn diagram of dysregulated genes in common in the two datasets. c. The molecular docking model of quercitrin with GJB2. d. The expression profiling of GJB2 in LUAD tissue and normal tissue. e. Patient populations were split into high expression and low expression groups by median value (Cutoff value = 418). The higher expression level of GJB2 was associated with worse overall survival in patients with LUAD.

**Figure 3.**
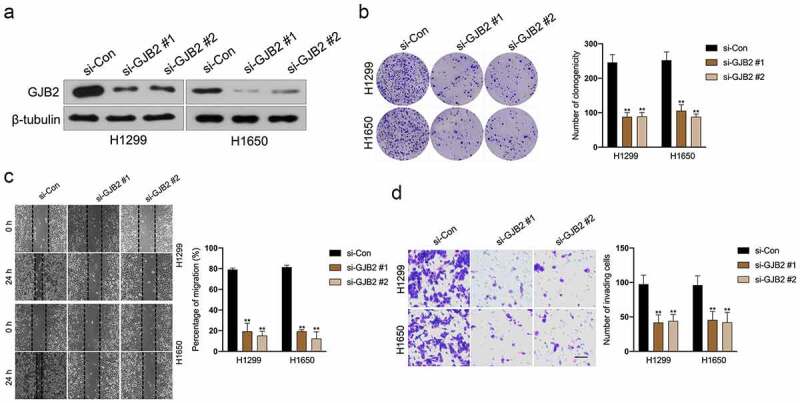
Deletion of GJB2 inhibits LUAD cells migration and invasion. a. H1299 and H1650 were transfected with si-GJB2 (#1 or #2). The expression of GJB2 was determined by Western blot. b. H1299 and H1650 were transfected with si-GJB2 (#1 or #2). The growth of LUAD cells was evaluated with a colony formation assay. c. Wound healing assay of GJB2 silencing H1299 and H1650 cell. d. Transwell invasion assay of GJB2 silencing H1299 and H1650 cell. Data represent the Mean ± SD for at least three independent experiments. **P< 0.01 compared with si-Con.

To determine potential prognostic factors for LUAD, dozens of dysregulated genes were obtained from the GSE18842 and GSE19804 databases with the aid of the R package ‘limma’ (Figure 2(a)). Using the R package ‘Venn’, a total of 27 genes (fold change > 2 and *P*< 0.05) were identified as upregulated in two GEO datasets (Figure 2(b)). In the proteins (ADAM12, PROM2, PSAT1, ANKRD22, KIF26B, CRABP2, THBS2, COL1A1, MMP11, LINC00673, HS6ST2, SIX1, ANLN, SULF1, COL10A1, CDCA7, CXCL14, CTHRC1, KIAA0101, TMPRSS4, COL11A1, SPP1, TOP2A, GREM1, GJB2, MMP1, MMP12) binding tests, the docking score of quercitrin and GJB2 was -12.97 kJ/mol and quercitrin exhibited the best binding activity toward GJB2 than other proteins. As shown in Figure 2(c) and Table 1, quercitrin forms three hydrogen bonds with active site residues of GJB2. Quercitrin interacted with active site residues TRP134 and SER138 of GJB2. Hence, we focused on GJB2, whose expression level is identified to be upregulated in LUAD [16]. Nevertheless, the potent role of GJB2 in LUAD cells’ growth and progression has not been explored. The expression pattern of GJB2 in LUAD was analyzed using GEPIA2 databases (http://gepia2.cancer-pku.cn/#index). The result revealed that GJB2 expression was upregulated in LUAD (Figure 2(d)). We also assessed the prognostic value of GJB2 in LUAD using the Kaplan Meier plotter (http://kmplot.com/analysis/index.php?p=service). Patient populations were split into high expression and low expression groups by the median value (Cutoff value = 418). As shown in Figure 2(e), the higher expression level of GIB2 was associated with poor overall survival of patients with LUAD (P value = 3.0e-5). To figure out whether GJB2 was related to the aggressive behaviors of LUAD cells, H1299 or H1650 cells were treated with siRNA GJB2 (si-GJB2 #1 or si-GJB2 #2). As shown in Figure 3(a), transfection of si-GJB2 lessened the expression of GJB2 in LUAD cells. Loss-function assay indicated that GJB2 deficiency profoundly attenuated the colony formation, migration, and invasion of LUAD cells *in vitro* (Figure 3(b,d)).

### GJB2 expression is reduced by quercitrin

**Figure 4.**
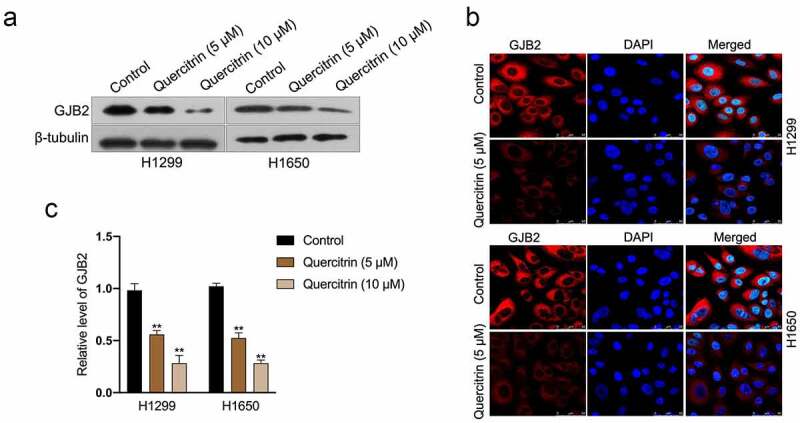
GJB2 expression is reduced by quercitrin. a. GJB2 expressions in quercitrin-treated H1299 and H1650 cells were determined by Western blot. b. The protein expression of GJB2 was determined by immunofluorescence. c. The mRNA levels of GJB2 in LUAD cells were determined using qRT-PCR. **P< 0.01 compared with control.

To evaluate the influences of quercitrin on GJB2, immunofluorescence staining, and Western blotting assay were carried out *in vitro*. In the immunoblotting assay, quercitrin inhibited the expression of GJB2 in LUAD cells in a dose-dependent manner (Figure 4(a)). The results of immunofluorescence staining ascertained that control cells exhibited stronger fluorescence staining of GJB2; on the contrary, a weaker signal of GJB2 expression was detected in quercitrin treated LUAD cells (Figure 4(b)). Subsequently, the effects of quercitrin on GJB2 expression at the transcriptional level were evaluated. As suggested in Figure 4(c), quercitrin dose-dependently inhibited the transcript levels of GJB2 in H1299 or H1650 cells.

### Reintroduction of GJB2 offset the inhibitory effect of quercitrin in LUAD cell

**Figure 5.**
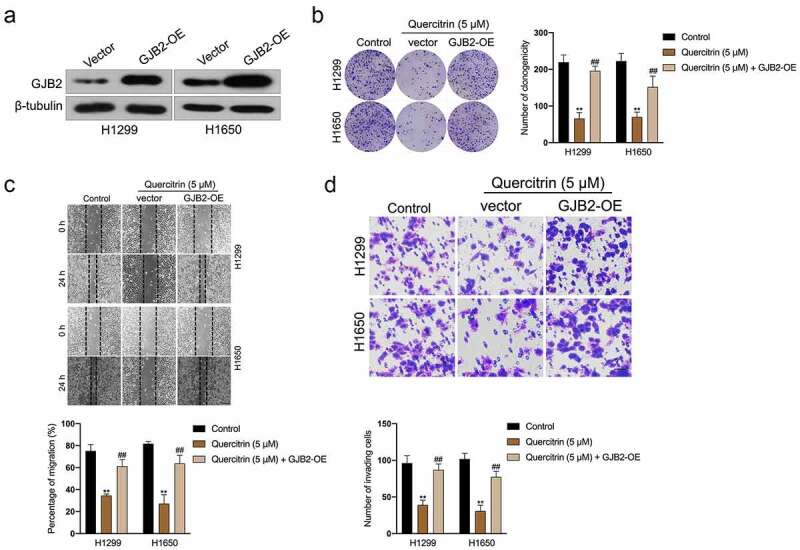
GJB2 is required for the ai-cancer effect of quercitrin in LUAD cell. a. GJB2 expressions in H1299 and H1650 cells transfected with GJB2-OE were determined by Western blot. b. Colony formation assay showed that the growth of H1299 and H1650 cells was increased after GJB2 overexpression in the presence of quercitrin. c-d. Wound healing and Transwell invasion assay showed that the migration and invasion abilities of H1299 and H1650 cells were increased after GJB2 transfection. Data represent the Mean ± SD for at least three independent experiments. **P< 0.01 compared with control, ^##^*P*< 0.01 compared with quercitrin.

To examine whether the tumor suppressor role of quercitrin in LUAD through the silencing of GJB2, pcDNA3.1 carrying GJB2 (GJB2-OE) was transfected into LUAD cells. The transfection efficiency was verified by Western blot (Figure 5(a)). Clonogenic formation experiments indicated that GJB2-OE rescued the growth of H1299 and H1650 cells prohibited by quercitrin (Figure 5(b)). Furthermore, wound healing and transwell assay proved that forced expression of GJB2 abrogated the inhibiting effects of quercitrin on the migration and invasive capacities of LUAD cells (Figure 5(c,d)). Collectively, GJB2 overexpression neutralizes the suppressive effect of quercitrin on LUAD cells.

## Discussion

Several components of Chinese herbal medicine can disturb the growth of cancerous cells both *in vitro* and *in vivo*, such as flavones, polysaccharides, phenols, and terpenoids. Flavonoids are dietary phenolic compounds found ubiquitously in plant foods such as fruits and vegetables. Most flavonoids have anti-inflammatory effects and anti-tumor activities [22,23]. Quercitrin, a glycosylated form of flavonoid compounds, exists widely distributed in nature. Emerging studies have disclosed that quercitrin exhibits anti-carcinogenic and anti-oxidant activities [24,25]. The pro-apoptotic effect of quercitrin in NSCLC has been reported previously [13,26]. Until now, the potential molecular mechanisms underlying the anti-metastasis effect of quercitrin in LUAD remain enigmatic.

First, we measured the inhibitory effect of quercitrin on LUAD cells growth. Based on the findings *in vitro*, we concluded that quercitrin exerted profoundly suppressive action on the cell viability of LUAD cells (H1299 and H1650) in both a dose-dependent manner and a time-dependent pattern. Similarly, quercitrin treatment led to dramatically reductive effects in the clonogenic activity of LUAD cells. Cell mobility, invasion, intravasation, extravasation, and metastasis formation are vital for cancer cells diffusion and distant metastases [27,28]. Hence, wound closure and Transwell assays were applied to measure the implications of quercitrin for the migration and invasiveness of LUAD cells *in vitro*. The results implied that quercitrin prohibited the LUAD cells migration and invasion *in vitro* in a dose-dependent fashion.

To figure out the underlying anticarcinogenic mechanism triggered by quercitrin in LUAD, two GEO databases (GSE18842 and GSE19804) were selected to screen the differential genes between lung cancer specimens and normal. The molecular docking method was used to explore the binding energy between quercitrin and these upregulated genes, and we observed that quercitrin exhibited the best binding activity toward GJB2 than other proteins. Moreover, we revealed that GJB2 was significantly upregulated in lung cancer, and patients with a higher level of GJB2 exhibits poor overall survival. To delineate the biological significance of GJB2, GJB2 silencing LUAD cells were constructed. The loss-of-function assays showed that deletion of GJB2 weakened the growth and invasion of LUAD cells. Early reports have been revealed GJB2 is involved in the progression of cancers. We strived to analyze the expression change of GJB2 in LUAD cells after being treated with quercitrin [29,30]. After LUAD cells were exposed to quercitrin, we corroborated that GJB2 protein expression and transcriptional level were significantly decreased.

Moreover, upregulation of GJB2 with the aid of pCDNA3.1-GJB2 plasmid canceled the inhibitory activity of quercitrin against LUAD cells, which indicates that GJB2 is the potential target for quercitrin. When GJB2 overexpressing LUAD cells were exposed to quercitrin, the suppressive profiles of quercitrin were diminished. All these observations suggested that the anti-cancer effect triggered by quercitrin in LUAD is attributed to the inhibition of GJB2. Our work comes with a few limitations worth noting. A previous study by Cincin, et al established that quercitrin has anti-proliferative and apoptotic effects on lung cancer cells through modulating the immune response [13]. Thus, follow-up studies are required to explore the impact of quercitrin on LUAD cells’ apoptosis. Furthermore, whether other flavonoids in *T. sinensis* leaves have an anti-cancer effect like quercitrin remains to be investigated. Moreover, it is necessary to clarify whether other flavonoids exert the anticancer effect by directly targeting GJB2.

## Conclusions

Altogether, our study manifested that quercitrin blocked the growth and metastatic traits of LUAD cells by inhibiting GJB2 (Fig. 6). There are further limitations to the study that need to be addressed. Further studies will be required to illuminate the molecular mechanism by which quercitrin regulates the transcript level of GJB2. The *in vivo* anticancer efficacy of quercitrin in tumor-bearing mice requires in-depth evaluation in the follow-up research.

## Supplementary Material

Supplemental Material

## Data Availability

The datasets used and/or analyzed during the current study are available from the corresponding author on reasonable request.
