# Supplementary material for: Quercitrin restrains the growth and invasion of lung adenocarcinoma cells by regulating gap junction protein beta 2
Source: Bioengineered. 2022 Feb 23;13(3):6126–35. doi: 10.1080/21655979.2022.2037372 (PMC8973705; doi:10.1080/21655979.2022.2037372)
Supplement: Supplemental Material [file KBIE_A_2037372_SM4552.docx]

**Animal experiments**

BALB/c nude mice (3-4 weeks) were purchased from Shanghai Experimental Animal Center (Chinese Academy of Sciences, Shanghai, China) and maintained in laminar flow cabinets under specific pathogen-free conditions. All operations were carried out according to protocols approved by the Animal Ethics Committee of the Zhengzhou University of Industrial Technology. For the xenograft tumor model, H1299 cells (1 × 10^7^) were subcutaneously injected into a single flank of each mouse. Tumor growth was examined every week, and tumor volume was calculated using the following equation: tumor volume = 0.5 × length × width^2^. When the tumor size reached around 100 mm^3^, mice were randomly divided into two groups (vehicle group and quercitrin group, n =3 in each group). The mice were treated with either 100 μl 0.5% carboxymethylcellulose sodium (vehicle group) or 100 mg/kg quercitrin (100 suspended in 0.5% carboxymethylcellulose sodium daily) *via* oral gavage. Five weeks after treatment, the mice were euthanized *via* CO_2_ asphyxiation, and tumor weights were measured.


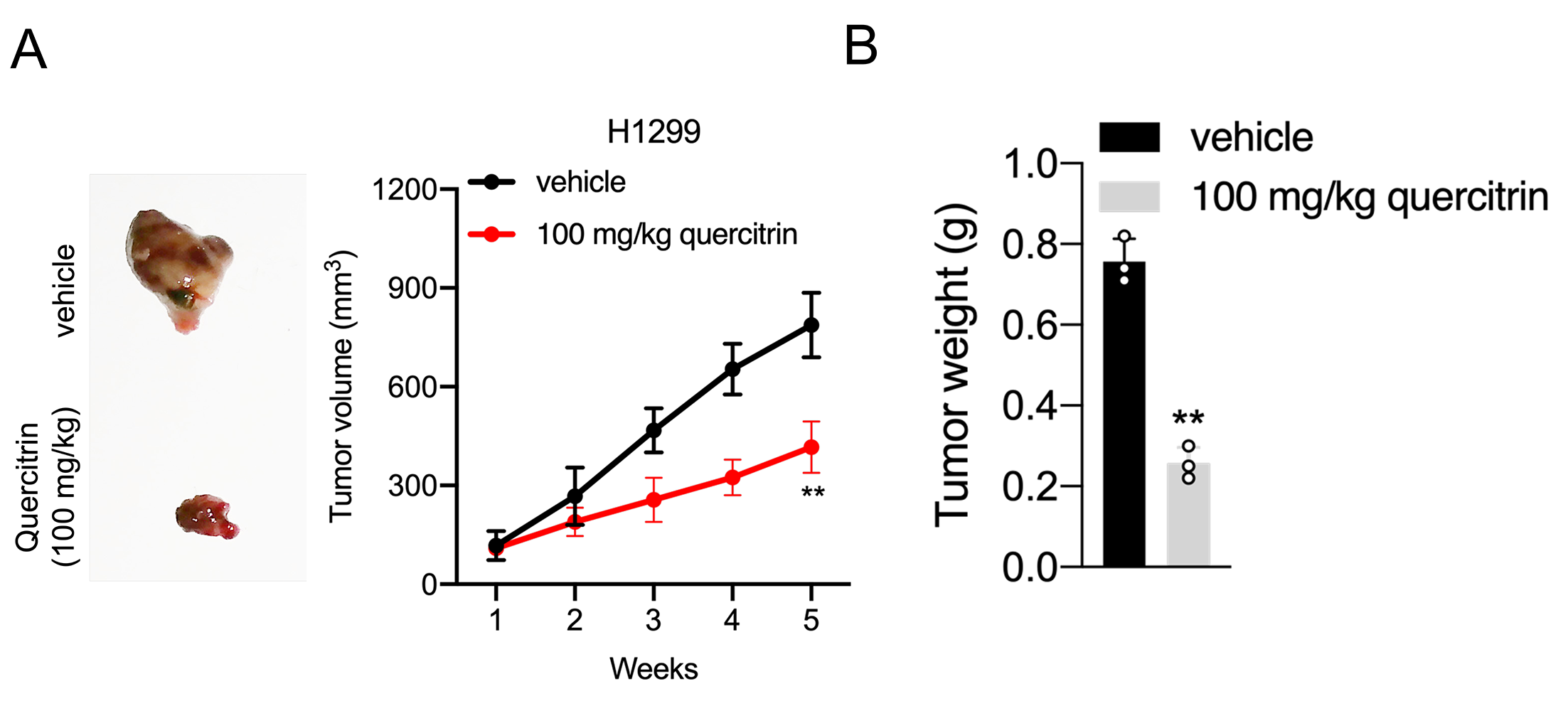


**Supplementary Figure 1. Quercitrin impairs tumor growth of** **H1299 cells *in vivo*. A**. The volume of H1299 xenograft tumors was significantly suppressed by 100 mg/kg quercitrin compared with the vehicle group. ^**^*P*<0.01 compared with vehicle. **B**. The weights of H1299 xenograft tumors were significantly suppressed by 100 mg/kg quercitrin compared with the vehicle group. ^**^*P*<0.01 compared with vehicle.
